# Comparing the Altis RepliGut Organoid System to MDCK Monolayers in Predicting the Oral Absorption of Lenalidomide

**DOI:** 10.3390/pharmaceutics17091140

**Published:** 2025-08-30

**Authors:** Cole S. Hudson, Jonathan Cheong, Jesse Yu, Eugene C. Chen, Laurent Salphati, Matthew R. Durk, Benjamin Lai, Karen Samy

**Affiliations:** 1 Department of Pharmacological & Pharmaceutical Sciences, University of Houston College of Pharmacy, Houston, TX 77004, USA; cole.hudson@bcm.edu; 2 Department of Drug Metabolism and Pharmacokinetics, Genentech, South San Francisco, CA 94080, USA; jonathac@gene.com (J.C.); yu.jesse@gene.com (J.Y.); chene28@gene.com (E.C.C.); salphati@gene.com (L.S.); durkm@gene.com (M.R.D.)

**Keywords:** intestinal organoids, MDCK cells, lenalidomide, drug permeability, oral bioavailability

## Abstract

**Background:** Predicting oral drug absorption in humans is critical during early drug development. Current in vitro systems to predict absorption (e.g., PAMPA and MDCK cells) are lacking for certain classes of drugs. Intestinal organoids are emerging as a promising alternative that offers several potential advantages. In this study, we utilized human intestinal organoid-derived monolayers to predict oral absorption of lenalidomide. **Methods:** Human jejunal organoids (RepliGut^®^) were cultured as monolayers on transwell plates and differentiated into intestinal epithelial cells. Lenalidomide permeability in the organoid system was compared with the permeability in the conventional Madin-Darby Canine Kidney cell (MDCK) monolayer system, as well as P-gp knockout, human P-gp overexpressing, and human BCRP overexpressing MDCK cells across a concentration range of 1 to 500 µM. Male Sprague Dawley rats were administered lenalidomide orally/intravenously, and concentrations in the serum, urine, and feces were measured and modeled in Phoenix WinNonlin. **Results:** Orally administered lenalidomide was well absorbed by rats at all doses (bioavailability = 68–120%). In the human jejunal organoid model, lenalidomide apparent permeability (Papp) was approximately 0.6 × 10^−6^ cm/s independent of the concentration used (1–500 µM). In contrast, lenalidomide Papp was significantly lower in gMDCK cell monolayers, approximately 0.2 × 10^−6^ cm/s. Additionally, lenalidomide was identified as a P-gp/BCRP substrate in intestinal organoids and gMDCK P-gp and BCRP overexpressing cells. **Conclusions:** Lenalidomide Papp was significantly lower in gMDCK monolayers than expected based on its high bioavailability. Our results suggest that organoid systems can better capture transporter and paracellularly mediated effects on drug permeability, which may allow for more accurate predictions of in vivo absorption.

## 1. Introduction

Bioavailability is an important pharmacokinetic parameter that describes the percentage of drug reaching the systemic circulation following oral administration. A high fraction absorbed across the gut epithelium is desirable to achieve higher and more reliable systemic concentrations. Therefore, it is valuable to predict the extent of human drug absorption using in vitro and/or in vivo systems to better position drug candidates during pharmaceutical development. In vitro systems are preferred for high-throughput evaluation of drug candidate permeability, reducing the cost of drug development and the number of animals used and accelerating the development of therapeutics.

Current in vitro permeability systems include parallel artificial membrane permeability assay (PAMPA), as well as immortalized cell lines such as Madin-Darby Canine Kidney (MDCK) and human colorectal adenocarcinoma (Caco-2) cells. PAMPA is a high-throughput screening tool using lipid-infused artificial membranes to predict cell permeability [1]. A major downside of this technique is that it does not capture transporter and paracellularly mediated effects on drug permeability. MDCK and Caco-2 cell monolayers are more physiologically relevant permeability models than PAMPA but still do not exactly mimic the physiological function of the intestinal epithelial cells (IECs). For instance, these immortalized cell monolayers have been reported to form significantly tighter junctions than IECs in vivo, which could underpredict the permeability of paracellularly permeating compounds [2,3,4,5]. Additionally, immortalized cell lines used for permeability assays express some transporters (e.g., P-gp and BCRP) at significantly different levels than IECs in vivo [6,7,8]. Finally, MDCK/Caco-2 permeability systems are composed of a single cell type that does not capture the complexity of the intestinal epithelia in vivo. These differences, among others, can lead to unreliable estimations of in vivo absorption. For example, a poor correlation (r^2^ = 0.38) was seen between MDCK apparent permeability (Papp) and human fraction absorbed for 97 drugs [9].

Recently, intestinal stem cell-derived organoid systems, such as RepliGut, have emerged as an in vitro organ model that offer a promising alternative to immortalized cell monolayers. Organoids are derived from primary intestinal stem cells that can differentiate into various cell types and be cultured as a monolayer that may be more representative of the human intestinal epithelia than MDCK cells or 3D organoids. The monolayer can include cell types such as goblet cells, enterocytes, enteroendocrine, and microfold cells (M cells) in the presence of Wnt, RankL, epidermal growth factor (EGF), or even intestinal tissue homogenate [10,11]. Intestinal organoids are highly physiological, possessing metabolic functions, drug transporters, and cell junctions that are more similar to IECs in vivo [12,13,14]. For example, human intestinal organoid-derived monolayers have been found to have comparable gene expression of drug-metabolizing and drug-transporting enzymes to that of the human small intestine [14,15]. Notably, intestinal organoids have been shown to express P-glycoprotein (P-gp), which can influence oral drug absorption [10].

**Figure 1:**
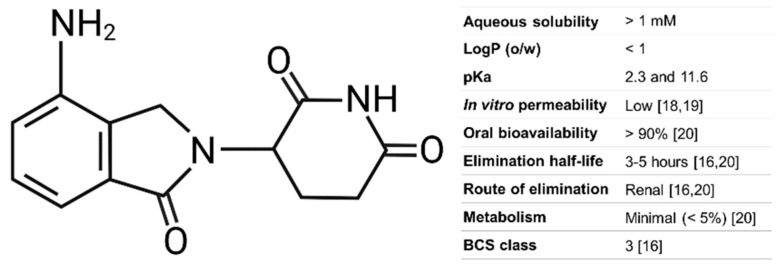
Lenalidomide structure and relevant properties in humans. The aqueous solubility and LogP value were obtained from PubChem. The pKa was computed by Chemaxon.

Lenalidomide is an immunomodulatory drug used for the treatment of various lymphomas [16]. It is a “molecular glue” compound that binds to cereblon (CRBN), the substrate receptor subunit of an E3 ubiquitin ligase complex. Lenalidomide binding alters the substrate specificity of this complex, resulting in the degradation of specific proteins that promote cancer cell survival (e.g., IKZF1/3). Its structure and relevant properties are shown in Figure 1. Lenalidomide’s pKa values of 10.2 and 2.3 suggest that it would be mostly unionized under physiological conditions in the small intestine but may become positively charged at stomach pH [16,17].

Lenalidomide is considered a BCS class 3 drug due to its high solubility but low permeability in MDCK cells and other traditional in vitro systems [16]. However, interestingly, lenalidomide shows rapid and extensive absorption in vivo, which may suggest that other transport mechanisms, which are not captured by current in vitro systems, may be involved in its absorption [18,19,20,21]. Lenalidomide has not been identified as a substrate for many common drug transporters, and its mechanism(s) of rapid in vivo absorption still remains largely unknown [22]. We chose lenalidomide as a representative compound with poor correlation between in vitro permeability and in vivo absorption. In this study, we utilized a commercially available human intestinal organoid-derived monolayer system, RepliGut^®^, to predict lenalidomide in vivo absorption compared with the traditional MDCK system. Additionally, we utilized P-gp-knockout MDCK cells (gMDCK) and gMDCK cells overexpressing human P-gp (gMDCK-MDR1) or BCRP (gMDCK-BCRP) efflux transporters [23] to provide insights into mechanistic differences between MDCK and intestinal organoid permeability systems.

## 2. Materials and Methods

### 2.1. Chemicals and Reagents

Lenalidomide was obtained from ThermoFisher Scientific (Waltham, MA, USA). Commercially available compounds were purchased from Sigma Aldrich (St. Louis, MO, USA). Dulbecco’s Modified Eagle’s Medium (4.5 g/L glucose, with L-glutamine), MEM amino acids, penicillin/streptomycin, TrypLE Select (10×), HEPES (100×), and Hank’s balanced salt solution were purchased from ThermoFisher Scientific (Waltham, MA, USA). HTS Transwell 96-well plates (0.4 um pore size, PET) were purchased from Corning Inc. (Corning, NY, USA). Culturing media for intestinal organoid-derived monolayer and coated transwell 96-well plates were provided by Altis Biosystems (Durham, NC, USA).

### 2.2. Animals

All animal pharmacokinetic experiments were carried out by WuXi (Shanghai, China). Animals were kept on a light-dark cycle and allowed to eat and drink *ad libitum*. Animals receiving oral doses were fasted overnight. Animals’ condition and behavior were monitored throughout the experiment for signs of pain and distress. We utilized only male rats because similar pharmacokinetics in both genders was previously reported and no dosage adjustment is recommended based on gender, in order to reduce the number of animals required [24].

### 2.3. Pharmacokinetic Study

Male Sprague Dawley rats (230–280 g) (n = 3 per group) were administered lenalidomide (0.1, 1, 2, 10, and 50 mg/kg). The vehicles for oral and intravenous dosing were 0.6% methylcellulose A15 + 0.2% Tween 80 in water and 10% DMSO + 60% propylene glycol + 30% water, respectively. Serum samples were obtained prior to administration (predose) and at 0.033, 0.083, 0.25, 0.5, 1, 2, 4, and 6 h postdose from the IV group and at 0.083, 0.25, 0.5, 1, 2, 4, 8, and 24 h from the oral groups. Approximately 250 μL was collected at each time point via a catheter at the femoral artery from each rat into prechilled commercial K2-EDTA tubes on wet ice. Samples were centrifuged (3200× *g* for 10 min at 4 °C) within one hour of collection and frozen at −60 °C or lower before analysis. Approximately 5 mL of whole blood was collected from additional blank animals into prechilled commercial K2-EDTA tubes on wet ice and pooled to serve as blank plasma for bio-analysis. Urine and feces were collected from the IV groups before dosing and from 0 to 8 and 8 to 24 h postdose. Animals were kept in metabolic cages to collect urine, feces, and bile at different periods after dosing. Wet ice was added during the whole collection process for urine and feces. Feces was homogenized in water, and the urine samples and feces homogenates were kept at −70 ± 10 °C until LC-MS/MS analysis. Pharmacokinetic parameters were calculated by non-compartmental methods, as described in Gibaldi and Perrier [25], using Phoenix^®^ WinNonlin^®^ version 8.5 (Certara L.P., Radnor, PA, USA). Following PO administration, bioavailability (%F) was determined for each animal by dividing the dose normalized area under the plasma concentration–time curve, extrapolated to infinity (AUCinf), obtained following each PO dose by the mean dose normalized AUCinf of the animals dosed by IV injection.

(1) $$Bioavailability=100\;\cdot \;\frac{{AUC}_{inf,\;oral}\;\cdot {Dose}_{IV}}{{AUC}_{inf,\;IV}\;\cdot {Dose}_{oral}}$$

The human equivalent dose (HED) was calculated based on the previously reported AUC after a typical dose in humans [26]. The percentage of lenalidomide excreted in the feces/urine in 24 h was calculated by multiplying the fecal weight/urine volume by its measured lenalidomide concentration and dividing by the total amount of lenalidomide administered.

(2) $$Percentage\;excreted\;in\;feces=100\;\cdot \;\frac{Feces\;weight\;\cdot Feces\;concentration\;}{Dose\;volume\;\cdot Dose\;concentration}$$

(3) $$Percentage\;excreted\;in\;urine=100\;\cdot \;\frac{Urine\;volume\;\cdot Urine\;concentration\;}{Dose\;volume\;\cdot Dose\;concentration}$$

### 2.4. Liquid Chromatography-Mass Spectrometry Conditions and Preparation of Standards and Samples

For the in vivo study, mass spectrometry detection of lenalidomide was accomplished using a SCIEX QTrap 6500+. Transition pairs used for quantification were as follows: lenalidomide (260.1 → 149.1 Da), labetalol (329.1 → 294.1 Da), indomethacin (357.9 → 139.0 Da), and loperamide (477.1 → 266.2 Da). Separation was achieved using a Waters Acquity UPLC system.

For urine samples, mobile phase A was 0.1% NH_4_OH and 2 mM NH_4_OAc in water/acetonitrile (v:v, 95:5), and mobile phase B was 0.1% NH4OH and 2 mM NH_4_OAc in acetonitrile/water (v:v, 95:5). Mobile phases were operated at the following gradient: initial, 1% B; 0–0.2 min, 1% B; 0.2–1, 30% B; 1–1.5, 100% B; 1.5–1.9, 100% B; 1.9–1.91, 1% B; and 1.91–2, 1% B. The column used was a Waters Acquity UPLC BEH C18 1.7 μm (2.1 × 50 mm) with flow rate of 0.6 mL/min and column oven temperature of 60 °C.

For plasma and feces samples, mobile phase A was 0.1% formic acid and 2 mM HCOONH_4_ in water, and mobile phase B was 0.1% formic acid and 2 mM HCOONH_4_ in acetonitrile/water (v:v, 95:5). Mobile phases were operated at the following gradient: initial, 1% B; 0–0.3 min, 1% B; 0.3–1.5, 30% B; 1.5–2.1, 95% B; 2.1–2.4, 95% B; 2.4–2.41, 1% B; and 2.41–2.5, 1% B. The column used was a Waters Acquity UPLC HSS T3 1.8 μm (2.1 × 50 mm) with a flow rate of 0.6 mL/min and a column oven temperature of 45 °C.

For the in vitro studies, compound detection was conducted using a SCIEX QTrap 6500. Transition pairs used for quantification were as follows: lenalidomide (260.1 →149.1 Da), nadolol (310.2 → 254.1 Da), labetalol (329.1 → 116.1 Da), metoprolol (268.2 → 116.1 Da), and amprenavir (506.2 → 245.1 Da). Propranolol (260.1 → 116.1 Da) was used as the internal standard. Mobile phase A was 0.1% formic in water, and mobile phase B was 0.1% formic acid in acetonitrile. Mobile phases were operated at the following gradient: starting at 5% acetonitrile, ramping to 98% acetonitrile from 0.5 to 0.8 min, remaining at 98% acetonitrile until 1.6 min, and dropping to 5% until the end of the 2 min run time. The column used was Kinetex 2.6 µm C18 100 Å (50 × 2.1 mm) with flow rate set at 1 mL/min and maintained at 40 °C during compound injection. Peak areas were quantified in MultiQuant software version 3.03 (SCIEX, Framingham, MA, USA).

For in vivo studies, feces samples were homogenized at a ratio of 1 g feces to 9 mL buffer. Samples were diluted into the linear assay range with a blank matrix. Calibration standards were prepared in a blank matrix. For in vitro studies, samples were diluted with the HEPES-buffered HBSS (with Ca^2+^ and Mg^2+^, 10 mM HEPES, pH = 7.4) solution and then diluted 1:3 with 50% water and 50% acetonitrile and 0.1% formic acid for injection. The mobile phase was spiked with 50 nM propranolol.

### 2.5. gMDCK Cell Culture

gMDCK cells were maintained in Dulbecco’s Modified Eagle’s Medium (DMEM) supplemented with Gibco’s MEM amino acids solution (1 X), penicillin (100 units/mL), streptomycin (100 mg/mL), and 10% fetal bovine serum and in a humidified, 37 °C, 5% CO_2_ environment as previously described [23]. Efflux transporter-expressing gMDCK cells, gMDCK-P-gp and gMDCK-BCRP, were cultured in the above-mentioned medium with addition of puromycin (2 µg/mL). All cells were passaged at approximately 80% confluency in T-175 flasks. For permeability assays, gMDCK, gMDCK-P-gp, and gMDCK-BCRP cells were seeded on 96 transwell plates (approximately 40,000 cells/well) and allowed to grow for 48 h in a 37 °C, 5% CO_2_ environment. Digitonin (10–40 mg/L) was included in some experiments to loosen tight junctions to explore paracellular permeability. TEER values were measured on the day of the experiment using an EVOM^TM^ Auto (World Precision Instruments, Sarasota, FL, USA).

### 2.6. Intestinal Organoid Culture from Altis Biosystems

Detailed protocols on coating of the RepliGut^®^ 96-well transwell, preparing growth/maturation media, and establishing human intestinal epithelial organoids are described in accordance with Altis Biosystems.

First, pre-coated RepliGut^®^ plates were washed once with sterile phosphate buffered saline (PBS). The PBS was then aspirated, and the wells were air-dried prior to cell seeding. Frozen intestinal epithelial cells (HISC-JEJ/D5) were cultured onto the 96-well RepliGut^®^ plate. Briefly, frozen cell fragments were thawed in a 37 °C water bath for 2 min. The cell fragments were transferred to a 15 mL conical tube and centrifuged at 600× *g* for 1 min. The pellet was washed once with PBS and centrifuged at 600× *g* for 1 min. The pellet was then dissociated with 150 μL of warm Cell Dissociation Solution (IN-CDS) with vigorous pipetting of the solution to mix the pellet. The cells were dissociated at 37 °C for 3 min and further strained into smaller cellular fragments with another mechanical dissociation using the pipette tip. The cell dissociation was neutralized by the addition of 10 mL of RepliGut^®^ Growth Medium (MED-RGM-200). An amount of 100 µL of the homogeneous cell suspension was then added to the apical compartment of each well. The transwell plate was cultured in a humidified CO_2_ incubator at 37 °C with growth media changed every 48 h. Once forming a monolayer, the intestinal epithelial cells were differentiated with the RepliGut^®^ Maturation Medium (MED-RMM-200). The intestinal epithelial cells were further cultured for 6 days before lenalidomide permeability studies (total culture time was ~10 days). TEER values were measured on the day of the experiment.

### 2.7. In Vitro Permeability Assay with Human Intestinal Organoid-Derived Monolayer and gMDCK Monolayer

Lenalidomide dosing solutions (1–500 µM) were diluted from DMSO stock (500 mM) with HEPES-buffered HBSS (with Ca^2+^ and Mg^2+^, 10 mM HEPES, pH 7.4) + 20 µM Lucifer yellow resulting in a final DMSO content of 0.2%. Lucifer yellow was measured fluorometrically (Ex/Em 428/532 nm) to assess tight junctions/paracellular transport. Permeability of lenalidomide was assessed bidirectionally in both in vitro models over three hours, and cell monolayers were collected at the end of the experiment.

Briefly, for assessing apical to basolateral (A to B) permeability, 150 uL of lenalidomide dosing solutions was added to apical side of the transwell, and 250 uL of HEPES-buffered HBSS without Lucifer yellow was added to basolateral side. For evaluating basolateral to apical (B to A) permeability, 250 uL of dosing solutions was added to the basolateral side, and 150 uL of HEPES-buffered HBSS without Lucifer yellow was added to the apical side. An amount of 10 µL was sampled from the dosing side of the transwell at the beginning and the end of the experiment. An amount of 25 µL was sampled from the receiver side at 1, 2, and 3 h. Nadolol, labetalol, metoprolol, and amprenavir were chosen as reference compounds due to their varying apparent permeabilities and affinity for efflux transporters.

Apparent permeability (P_app_) was calculated according to the following equation: P_app_ = (dQ/dt)/(A * C), where dQ/dt is the amount of drug that crosses the membrane per unit of time, A is the surface area of the membrane through which the drug is permeating (0.143 cm^2^), and C is the initial concentration added to cells. The efflux ratio was calculated according to the following equation:

(4) $$Efflux\;ratio=\;\frac{P_{app}\;basolateral\;to\;apical}{P_{app}\;apical\;to\;basolateral}$$

### 2.8. Measuring Transporter Expression in the Intestinal Organoid-Derived Monolayer with Real-Time Polymerase Chain Reaction (qPCR)

Briefly, after 6 days of culturing in maturation medium, the intestinal organoid-derived monolayer was washed once with PBS (without Ca^2+^ and Mg^2+^) and dislodged from the transwell membrane with a pipet tip. Monolayers from individual wells were collected and centrifuged at 500× *g* for 3 min. RNA samples were extracted and purified using the RNeasy kits (Qiagen, Hilden, Germany), in accordance with the manufacturer’s protocol. An amount of 2 µg of RNA was then used to synthesize complementary DNA (cDNA) with the Superscript IV VILO master mix (ThermoFisher Scientific) according to the manufacturer’s instructions. Forward/reverse primers and probes of the intestinal transporters were custom designed onto the 384-well Taqman array plate (ThermoFisher Scientific). An amount of 10 µL of the qPCR mixture consisting of 10 ng of cDNA samples, Taqman multiplex master mix (ThermoFisher Scientific), GADPH-JUN (GAPDH as housekeeping gene, with JUN as the reporting fluorescent dye [catalog # 4485713, Applied Biosystems, Waltham, MA, USA]), and nuclease free water was loaded into the wells of the Taqman array plate. Real-time PCR was then performed by using the QuantStudio 6 Flex Real-Time PCR system (ThermoFisher Scientific) in accordance with the Taqman multiplex master mix. Levels of various transporters were reported as a ratio to GAPDH levels (no units).

### 2.9. Statistical Analysis

All statistical analyses were conducted in GraphPad Prism version 10.4.1 (GraphPad Software, Boston, MA, USA). A multiple t-test with correction for multiple comparisons using the Holm-Sidak method was used where appropriate. We did not take any steps for small sample size adjustment (*n* = 3) nor check for normality.

## 3. Results

### 3.1. Lenalidomide Pharmacokinetics in Rats

**Figure 2:**
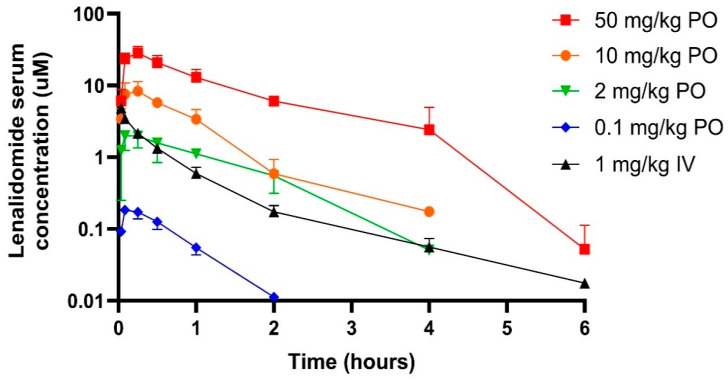
Lenalidomide serum pharmacokinetic profiles in rats following intravenous and oral administration. Observations represent the mean serum concentration ± SD (N = 3 male rats per group).

Lenalidomide (1 mg/kg) was delivered by IV bolus (Figure 2) to provide an AUC (2.34 µmol×hr/L) for bioavailability calculations and to estimate renal clearance (24 mL/min/kg) (Table 1). Lenalidomide was mostly excreted unchanged in the urine (87%). Interestingly, lenalidomide was present in the feces (3.2% of dose) following IV administration. The human equivalent dose (HED) was approximately 11 mg. A typical dose in humans is 25 mg [16].

Lenalidomide was delivered orally at doses of 0.1, 2, 10, and 50 mg/kg (Figure 2). The bioavailability of the low doses was greater than 100%, while the bioavailability of the high doses was less than 100% (Table 1). The half-life at the highest oral dose was notably longer (2.7 h) than the half-life at the lower doses. Tmax remained relatively constant (~0.5 h) as did fecal recovery (~12% of dose). Urinary recovery was approximately 45% for all oral doses. HEDs of 0.1, 2, 10, and 50 mg/kg were approximately 1.4, 26, 73, and 376 mg, respectively.

### 3.2. Lenalidomide Permeability in Intestinal Organoids and gMDCK Cells

**Figure 3:**
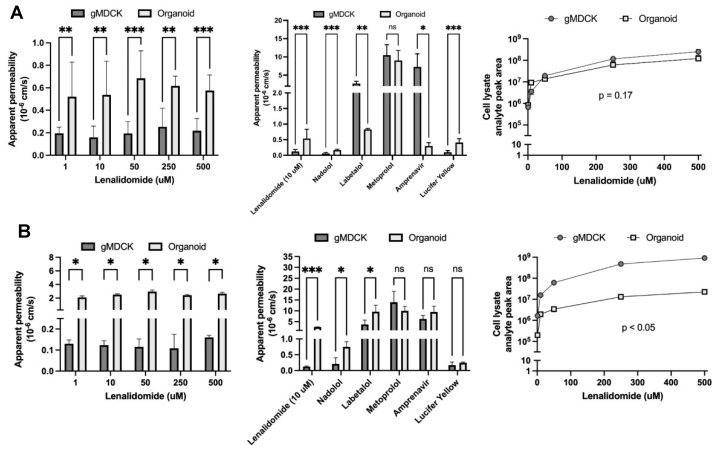
Apparent permeability and intracellular accumulation of lenalidomide and reference compounds in human intestinal organoid-derived and gMDCK monolayers. (**A**) Apparent permeability of compounds dosed in apical side (apical → basolateral) and (**B**) basolateral side (basolateral → apical). gMDCK = gMDCK monolayer and organoid = RepliGut intestinal organoid monolayer system. Data reported as mean ± SD. For gMDCK cells N = 3 biological replicates for permeability studies and *n* = 3 technical replicates for intracellular accumulation. For organoids, *n* = 3 technical replicates. *** *p* < 0.001, ** *p* < 0.01, * *p* < 0.05, ns *p* > 0.05.

The apical to basolateral apparent permeability of lenalidomide was significantly higher in intestinal organoids compared with gMDCK monolayers at all concentrations (*p* < 0.01) (Figure 3A). The apparent permeability of both nadolol and Lucifer yellow (paracellular permeability markers) was also higher in the intestinal organoid-derived monolayers than in gMDCK monolayers (*p* < 0.001). The permeability of known efflux transporter substrates, labetalol and amprenavir, was significantly lower in intestinal organoids (*p* < 0.05). Metoprolol showed similar permeability in intestinal organoids and gMDCK cells (*p* > 0.05). These findings are summarized in Table 2. When lenalidomide was dosed apically, there was no significant difference between the accumulation of lenalidomide in the cellular compartments of either intestinal organoid-derived monolayers or gMDCK monolayers (*p* = 0.17).

The basolateral to apical permeability of lenalidomide was significantly higher in intestinal organoids compared with gMDCK cells at all concentrations (Figure 3B) (*p* < 0.05) and the accumulation of lenalidomide was greater in gMDCK cells than organoids (*p* < 0.05). The permeability of nadolol and labetalol (known efflux transporter substrates) was significantly higher in intestinal organoids (*p* < 0.05) in the basolateral to apical direction. The permeability of metoprolol, amprenavir, and Lucifer yellow was similar in intestinal organoids and gMDCK cells with basolateral dosing (*p* > 0.05). Nadolol, labetalol, and amprenavir all showed notable efflux (efflux ratio > 4) by intestinal organoids, while metoprolol showed no efflux (efflux ratio ~ 1).

### 3.3. Lenalidomide Efflux by P-Gp/BCRP

**Figure 4:**
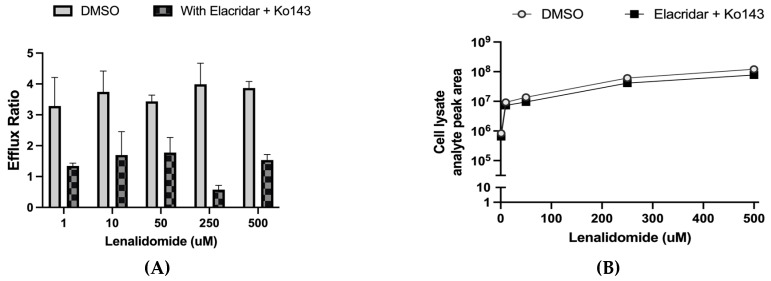
Lenalidomide efflux by P-gp/BCRP in organoids is notable (**A**) but does not affect intracellular accumulation (apical dosing) (**B**). Data reported as mean ± SD (*n* = 3 technical replicates). Efflux ratio = (basolateral → apical apparent permeability/apical to basolateral apparent permeability). RepliGut intestinal organoid system.

**Figure 5:**
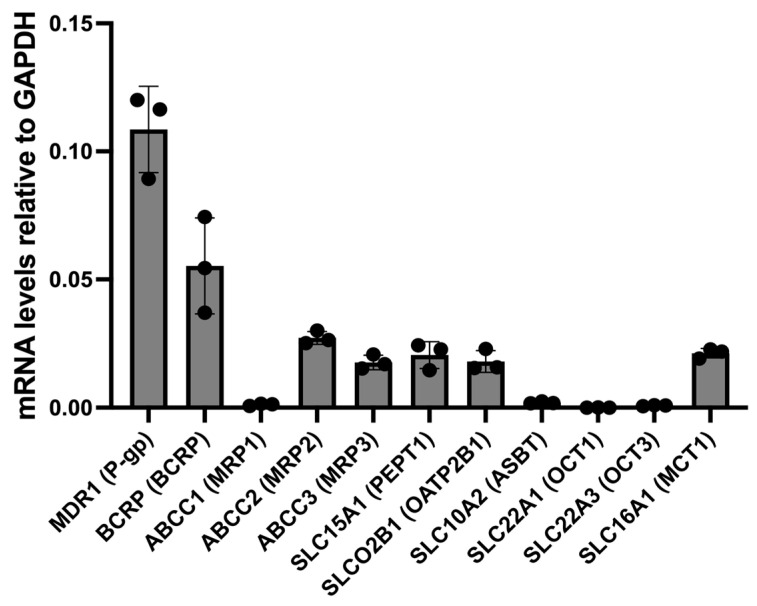
mRNA sequencing of various drug transporters in RepliGut intestinal organoid system. Data shown as mean ± SD (*n* = 3 technical replicates).

Lenalidomide showed notable efflux by intestinal organoids (efflux ratio 3–4) at all concentrations (Figure 4A). Addition of P-gp and BCRP inhibitors, elacridar (5 μM) and Ko143 (1 μM), reduced the efflux ratio to approximately 1. Interestingly however, intracellular lenalidomide concentrations in intestinal organoids were similar with and without elacridar (Figure 4B). Transporter expression levels in the intestinal organoid-derived monolayer are shown in Figure 5.

No efflux of lenalidomide was observed in gMDCK cells (efflux ratio = 0.62) (Table 3). However, lenalidomide showed notable efflux by both gMDCK P-gp and BCRP-overexpressing cells, with efflux ratios of 14 and 16, respectively, suggesting that lenalidomide is a substrate for both transporters.

## 4. Discussion

During the early stages of new drug development, predicting oral drug absorption is critically important. Current in vitro systems used to predict drug absorption have been shown to be inaccurate for certain compounds. This may be due to their inability to accurately capture transporter-mediated and paracellularly mediated effects on drug permeability. Intestinal organoids are emerging as an exciting new technology that has the potential to more accurately reflect the intestinal epithelium, especially transporter and paracellularly mediated effects on drug permeability.

In this study, lenalidomide showed rapid, extensive absorption following oral administration in rats at and below the human equivalent dose (2 mg/kg). The >100% bioavailability at low doses suggests complete absorption of lenalidomide. At the higher doses, the bioavailability dropped to ~60%. Since lenalidomide has a high solubility (at least 1 mM), this drop in bioavailability is likely not due to drug precipitation and could suggest saturation of an active uptake mechanism. Additionally, the longer half-life at the highest oral dose compared with the IV half-life suggests an absorption rate limited clearance (flip-flop kinetics). Together, these findings are consistent with extensive oral absorption due to a saturable drug uptake mechanism. Following IV administration, lenalidomide was predominantly excreted unchanged in urine, consistent with the previous literature [20]. However, lenalidomide presence in the feces following IV administration suggests that either biliary excretion or direct intestinal excretion by intestinal efflux transporters may be involved. Additionally, the renal clearance being higher than glomerular filtration rate (GFR) multiplied by the drug fraction unbound (fu) (4.5 mL/min/kg) indicates that lenalidomide undergoes active renal secretion, which has been reported by others [20].

In contrast to our in vivo findings, lenalidomide permeability in the conventional gMDCK monolayer assay was low under all conditions, independent of concentration (Papp ~ 0.2 × 10^−6^ cm/s). This would result in low, inaccurate prediction of lenalidomide in vivo oral absorption. Notably, significant lenalidomide accumulation was observed in gMDCK cell monolayers. In contrast to the conventional gMDCK assay, lenalidomide permeability was significantly higher in intestinal organoid-derived monolayers at all concentrations with a Papp around 0.6 × 10^−6^ cm/s. Intracellular accumulation in organoids was significantly lower (approximately 10-fold) in the basolateral to apical direction. The higher permeability and lower lenalidomide cellular accumulation by organoids under these conditions are consistent with the activity of a drug transporter(s), transporting lenalidomide into and/or out of the organoids. This is also consistent with our in vivo findings of saturable lenalidomide absorption. However, lenalidomide has not been demonstrated as a substrate for many common drug uptake transporters [22]. This has led us, and others, to postulate that there is an unspecified transporter contributing to lenalidomide permeability. Future inhibition assays would be valuable to identify uptake transporters contributing to lenalidomide permeability.

Transepithelial electrical resistance (TEER) values for gMDCK cells were much higher than the human intestinal organoid-derived monolayer. The gMDCK TEER values were ~4000 Ω∙cm^2^, compared with ~1600 Ω∙cm^2^ measured in the RepliGut intestinal monolayer (Appendix A). TEER values were generally consistent across replicate organoid wells. In vivo, the TEER values of the human small intestine are reported to be only 50–100 Ω∙cm^2^ [27]. In this way, intestinal organoids may be more representative of the cell junction tightness (and paracellular transport) observed in vivo. Less restrictive tight junctions allow higher paracellular permeability of compounds that are capable of passing between cells (i.e., paracellularly transported compounds). The transport mechanisms of lenalidomide are largely unknown, but it has the properties of a paracellularly transported compound: low molecular weight and hydrophilicity (Appendix B). Additionally, the apparent permeability of both nadolol and Lucifer yellow was significantly higher in organoids, which are believed to permeate paracellularly [28,29]. Therefore, our findings support that in addition to transporter effects, the higher paracellular drug permeability in organoids may be contributing to the higher observed lenalidomide permeability compared with gMDCK monolayers.

Notable efflux of lenalidomide was observed in intestinal organoids (efflux ratio 3–4, independent of concentration). The addition of P-gp/BCRP inhibitors, elacridar and Ko143, reduced lenalidomide efflux (efflux ratio ~1), suggesting little to no efflux. Similarly, we also observed efflux of lenalidomide in P-gp and BCRP-overexpressing gMDCK cells. This contradicts findings by others reporting that lenalidomide is not a BCRP substrate [16,22]. Potentially, this is due to different systems to assess BCRP interaction; we utilized gMDCK cells overexpressing BCRP, while the previous study utilized vesicles with BCRP [22]. Taken together, our findings indicate that lenalidomide is a substrate of both P-gp and BCRP and that these transporters are responsible for the majority of lenalidomide efflux. Although efflux of lenalidomide by organoids was limited with the addition of both elacridar and Ko143, intracellular lenalidomide accumulation remained similar. No efflux was observed in the conventional gMDCK monolayer assay. Taken with our other findings, this may suggest that an uptake transporter is transporting lenalidomide into the cell compartment.

We utilized several reference compounds of known permeability to increase the applicability of our findings to drugs of different classes and provide more consistent, reproducible results. Nadolol is a low-permeability drug and is known to be a substrate for efflux transporters (e.g., P-gp) but interestingly showed higher apical to basolateral permeability in the organoid system than gMDCK cells, potentially due to the less restrictive tight junctions. Labetalol and amprenavir showed lower permeability in organoids, most likely due to efflux transporter activity. Metoprolol, which is not an efflux transporter substrate, showed similar permeability in intestinal organoids and gMDCK cells.

Finally, an unwanted artifact of in vitro permeability systems is the formation of an unstirred water layer (UWL) that reduces the apparent permeability of compounds. It is reported that shaking the cell plate can reduce the thickness of the unstirred water layer and therefore its effects on drug permeability [30]. In this study, we explored shaking gMDCK cells during the permeability assay, but this did not significantly change permeability of lenalidomide or any reference compounds tested, including Lucifer yellow (Appendix C). Furthermore, we investigated the use of a sink condition in the gMDCK permeability assay by incorporating 4% bovine serum albumin in the receiver side to mimic in vivo-like conditions where the unbound lenalidomide is removed upon absorption. This maintains a concentration gradient in the in vitro permeability assay more closely simulating the in vivo absorption. However, this did not increase the apparent permeability of lenalidomide in the gMDCK assay. Therefore, we believe there may be other optimal conditions needed for assessing permeability of compounds like lenalidomide in an in vitro setting.

Limitations of this study include the use of only one conventional cellular permeability assay (gMDCK). The generalizability of the findings could be increased by including other cell permeability systems such as PAMPA and Caco-2. Additionally, other transporter(s) suggested to be involved in lenalidomide permeability could not be identified. Future experiments should focus on identifying uptake transporters that could contribute to lenalidomide permeability. Moreover, we did not investigate the potential variations in cellular diversity within the intestinal organoid monolayer during the differentiation stage, which may influence permeability. Integrating proteomic analyses on transporter expression with single-cell sequences on the intestinal organoid model may provide a more comprehensive understanding of this sophisticated model. Finally, application of mechanical shaking and a bovine serum albumin sink condition with the human intestinal organoid-derived monolayer may provide a more accurate prediction of lenalidomide intestinal permeability.

## 5. Conclusions

Lenalidomide permeability could not be accurately captured by the conventional in vitro MDCK monolayer system, failing to predict its extensive in vivo absorption. Our results suggest that paracellular and active transport could be involved in lenalidomide absorption and that organoid systems have more potential to capture transporter-mediated and paracellularly mediated effects on permeability, allowing more accurate prediction of in vivo absorption. The potential effect of uptake transporters on lenalidomide absorption needs further investigation.

## Figures and Tables

**Table 1:** Lenalidomide pharmacokinetic parameters in rats.

Dose	AUC (hr×µmol/L)	BA (%)	T_1/2_ (hr)	C_max_ (µM)	T_max_ (hr)	Fecal Recovery (% of Dose)	Urinary Recovery (% of Dose)	CL_R_ (mL/min/kg)	Human Equivalent Dose (mg)
1 mg/kg IV	2.34	100	1.2	4.9	0.03	3.2	87	24.0	11
0.1 mg/kg PO	0.29	122	0.87	0.20	0.42	12	47		1.4
2 mg/kg PO	5.44	116	1.4	1.6	0.33	12	45		26
10 mg/kg PO	15.4	66.0	1.4	8.4	0.42	10	48		73
50 mg/kg PO	79.4	67.9	2.7	29	0.50	16	43		376

N = 3 rats per group (males). Parameters were estimated using non-compartmental analysis in Phoenix WinNonlin. Human equivalent dose calculated based on previously reported AUC in humans after a 25 mg dose using a molecular weight of 259 g/mol (5.38 µmol×hr/L) [20]. AUC, area under concentration time curve from time 0 to last time concentration measured (calculated by Phoenix). Renal clearance (CL_R_) was estimated based on the amount excreted in the urine and AUC. BA, bioavailability.

**Table 2:** Main route of absorption, human fraction absorbed, and apparent permeability of lenalidomide and reference compounds in gMDCK cells and RepliGut human jejunal organoid-derived monolayers.

Compound	Main Route of Absorption	LogP	gMDCK Papp	Organoid Papp	Efflux Substrate	Human Fa
Lenalidomide (500 µM)	Unknown	−0.40	0.21	0.58	Yes	90% [21]
Nadolol	Paracellular	0.81	0.09	0.17	Yes	57% [9]
Labetalol	Both	1.8	3.0	0.85	Yes	95% [9]
Metoprolol	Transcellular	1.8	13	10.10	No	98% [9]
Amprenavir	Transcellular	2.2	8.5	0.35	Yes	Unknown

Papp, apparent permeability (×10^−6^ cm/s). For gMDCK cells, *n* = 3 biological replicates. For organoids, *n* = 3 technical replicates. Fa, fraction absorbed. LogP values were obtained from PubChem.

**Table 3:** Lenalidomide efflux by various gMDCK cell types and intestinal organoids.

Monolayer	Papp A to B	Papp B to A	Efflux Ratio
Human jejunal organoids	0.58	2.64	4.60
Human jejunal organoids + elacridar + Ko143	0.52	0.80	1.53
gMDCK	0.21	0.13	0.62
gMDCK-BCRP	0.15	2.4	16
gMDCK-MDR1	0.20	2.8	14

Each observation represents the average of at least two technical replicates. Papp, apparent permeability (×10^−6^ cm/s); A to B, apical to basolateral; B to A, basolateral to apical; efflux ratio, Papp B to A/Papp A to B.

**Figure A1:**
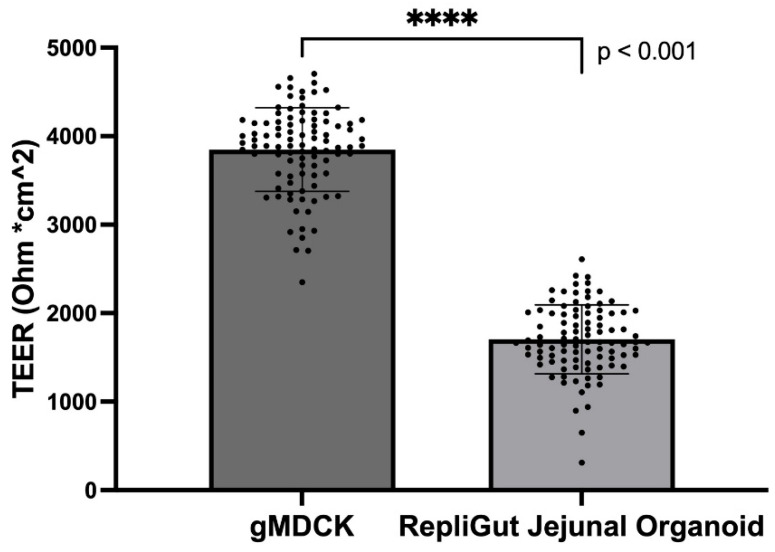
TEER Values in 96-Well Plate RepliGut Organoid System vs. gMDCK Cells. **** *p* < 0.001. Each group consists of *n* = 96 technical replicates. TEER was measured on the day of the experiment.

**Figure A2:**
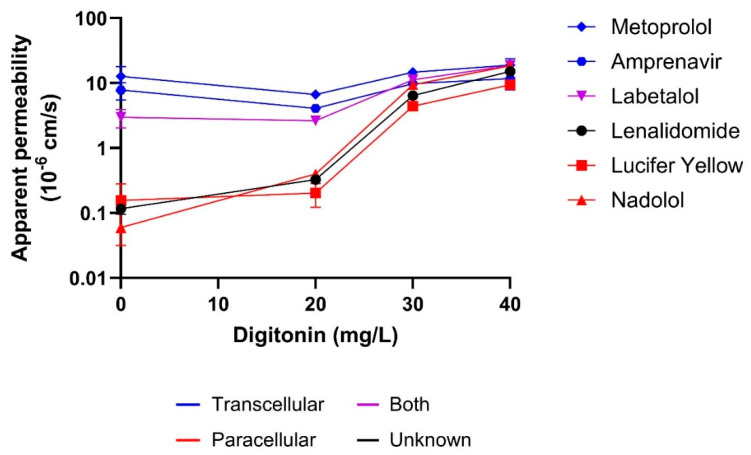
Apparent Permeability of Lenalidomide and Reference Compounds in gMDCK Monolayers with Adjuvant Digitonin Treatment. Each group consists of at least *n* = 3 technical replicates.

## Data Availability

The data presented in this study are available on request from the corresponding authors.

## Appendix C

**Table A1:** Lenalidomide Permeability in gMDCK Cells under Various Attempts to Mimic Physiological Conditions.

Lenalidomide (uM)	10	50	250	500
Typical assay conditions	0.19	0.22	0.24	0.24
FaSSIF	0.27	0.28	0.17	0.13
FeSSIF	0.07	0.14	0.10	0.14
4% BSA in receiver compartment	0.12	0.16	0.15	0.15
Shaking	0.03	0.03	0.14	0.21

Each observation is the average of at least three technical replicates. FaSSIF, fasted state simulated intestinal fluid; FeSSIF, fed state simulated intestinal fluid; BSA, bovine serum albumin (to mimic serum proteins); Shaking, shaking transwell plate at 1000 rpm.
